# Dental arch spatial changes after premature loss of first primary molars: a systematic review and meta-analysis of split-mouth studies

**DOI:** 10.1186/s12903-023-03111-x

**Published:** 2023-06-28

**Authors:** Jingzi Zhao, Hua Jin, Xiaoning Li, Xiurong Qin

**Affiliations:** 1 Department of Orthodontics, East Hospital District, Jinan Stomatological Hospital, Jinan, Shandong Province China; 2 Department of Stomatology, Harbin Institute of Technology Hospital, Harbin, Heilongjiang Province China; 3 Department of Endodontics, Yantai Stomatological Hospital Affiliated to Binzhou Medical College, Yantai, Shandong Province China; 4 Department of Pediatric Dentistry, Jinan Stomatological Hospital, Jinan, Shandong Province China

**Keywords:** Space loss, Premature tooth loss, Primary first molar, Spatial changes, Space maintainer

## Abstract

**Background:**

This study aimed to evaluate spatial changes in dental arches resulting from premature loss of first primary molars and assess the necessity of a space maintainer.

**Methods:**

We searched the electronic databases PubMed, Cochrane Library, ClinicalTrials, and EMBASE. Split-mouth studies involving unilateral premature loss of a primary first molar were included. Quality assessment of selected studies made use of the ROBINS-I tool. Mean space differences were calculated for the D + E and D spaces, arch width, arch length, arch perimeter.

**Results:**

Of the 329 studies considered, 11 split-mouth studies were selected, including 246 cases in the maxilla and 217 in the mandible from 477 individuals aged 5–10 years. Over the medium-term follow-up period (6–24 months), space loss was 0.65 mm in the maxillary D + E (MD 0.65, 95% CI 0.15–1.16, P = 0.01), 1.24 mm in the mandibular D + E (MD 1.24, 95% CI 0.60–1.89, P < 0.01), and 1.47 mm in the mandibular D (MD 1.47, 95% CI 0.66–2.28, P < 0.01). There was no significant change in arch width, length, or arch perimeter between the initial and follow-up examinations (P > 0.05).

**Conclusions:**

After premature loss of first primary molars, space can be lost, but the amount of loss would not affect arch width, length, or arch perimeter over the 6–24 months follow-up period.

**Supplementary Information:**

The online version contains supplementary material available at 10.1186/s12903-023-03111-x.

## Introduction

The concept of premature loss of primary teeth has been defined as exfoliation on the arch more than 12 months prior to the normal period of permanent tooth eruption [1, 2], which exceeds normal variability of the exfoliation sequences of temporary teeth. Premature loss of primary molars is usually caused by early extraction due to caries and/or failed pulp therapy, which may cause migration of adjacent teeth, space loss, crowding and impaction or dislocation of the permanent teeth, leading to the need for complex orthodontic treatment [1, 3–6]. In 1887, Davenport [7] described the concept of space loss caused by premature loss of primary teeth, and this problem has been studied since then. In 1998, Lin and Chang were the first to quantify the amount of space loss caused by premature loss of primary molars [8]. Since then, research on space changes following premature loss of primary teeth has included cross-sectional and longitudinal studies. At present, there are extensive clinical studies in the literature describing space changes resulting from premature loss of primary molars, including but not limited to the direction of the space change, the amount of space loss, and the need for space maintenance [9–12].

There are differences between the maxilla and mandible in terms of the space changes that occur following premature loss of primary first molars. Some studies have reported that space loss in the mandible is greater than that in the maxilla [13], while others have claimed the opposite [14]. Studies have shown more than one cause of space loss after premature loss of the primary first molar. Lin’s research showed that distal movement of the primary canine could occur after premature loss of the primary first molar on the maxilla and mandible [8], and mesial movement of permanent molars or tilting of the primary second molar did not occur [15]. Love and Adams [16] found that space loss caused by mesial movement of posterior teeth was higher than that caused by distal movement of anterior teeth, especially in the maxilla. In addition, some studies showed that space loss occurred in the maxilla due to mesial movement of the molars and mandible resulting from distal movement of the anterior teeth [17–19]. One study reported that premature loss of the primary mandibular first molar mainly results in distal drifting of the primary mandibular canine, while in the maxilla, mesial drifting of the primary second molar into the extraction space is more common [13].

Space maintenance is considered to play an important role in maintaining the integrity of the dental arch after premature loss of primary teeth [20]. Choonara [21] reported that many orthodontic cases involving crowding and insufficient space in permanent dentition could have been prevented or mitigated if the dentist had maintained sufficient space during the initial treatment of mixed dentition. Although there is little debate about the need for a space maintainer after premature loss of the primary second molar, there is disagreement about the need after premature loss of the primary first molar [15]. Some scholars believe that premature loss of the primary first molar will lead to loss of space, so the use of space maintainers becomes necessary [4, 7, 15]. However, others believe that arch length does not change after premature loss of the primary first molar, so the use of a space maintainer is unnecessary [13, 22, 23]. Some with views between these two extremes suggest that use of a space maintainer following premature loss of a primary first molar should be based on the dentist’s experience and the patient’s orofacial condition [24–26]. The purpose of this study was to evaluate dental arch spatial changes following premature loss of first primary molars and assess the need for a space maintainer.

**Table 1:** Search strategy

Database	Key Words	Results
PubMed	((((primary first molar ) OR (primary maxillary first molar ) OR (primary mandibular first molar))) AND (((tooth migration) OR (tooth drift)OR (mesial movement) OR (distal movement) OR (space loss) OR (arch changes)))) AND (((premature tooth loss ) OR (premature loss) OR (tooth loss) OR (tooth extraction) OR (tooth exfoliation))) Last update posted on or before 09/15/2022	100
Web of Science	((((primary first molar ) OR (primary maxillary first molar ) OR (primary mandibular first molar))) AND (((tooth migration) OR (tooth drift)OR (mesial movement) OR (distal movement) OR (space loss) OR (arch changes)))) AND (((premature tooth loss ) OR (premature loss) OR (tooth loss) OR (tooth extraction) OR (tooth exfoliation))) Last update posted on or before 09/15/2022	63
Cochrane Libray	((((primary first molar ) OR (primary maxillary first molar ) OR (primary mandibular first molar))) AND (((tooth migration) OR (tooth drift)OR (mesial movement) OR (distal movement) OR (space loss) OR (arch changes)))) AND (((premature tooth loss ) OR (premature loss) OR (tooth loss) OR (tooth extraction) OR (tooth exfoliation))) in Title Abstract Keywords Last update posted on or before 09/15/2022	77
EMBASE	(‘primary first molar’ OR (primary AND first AND (‘molar’/exp OR molar)) OR ‘primary maxillary first molar’ OR (primary AND (‘maxillary’/exp OR maxillary) AND first AND (‘molar’/exp OR molar)) OR ‘primary mandibular first molar’ OR (primary AND mandibular AND first AND (‘molar’/exp OR molar))) AND (premature AND tooth AND loss OR (premature AND loss) OR (tooth AND loss) OR (tooth AND extraction) OR (tooth AND exfoliation)) AND (space AND loss OR (tooth AND migration ) OR (tooth AND drift ) OR (mesial AND movement) OR (distal AND movement) OR (arch AND changes)) Last update posted on or before 09/15/2022	87

**Fig. 1:**
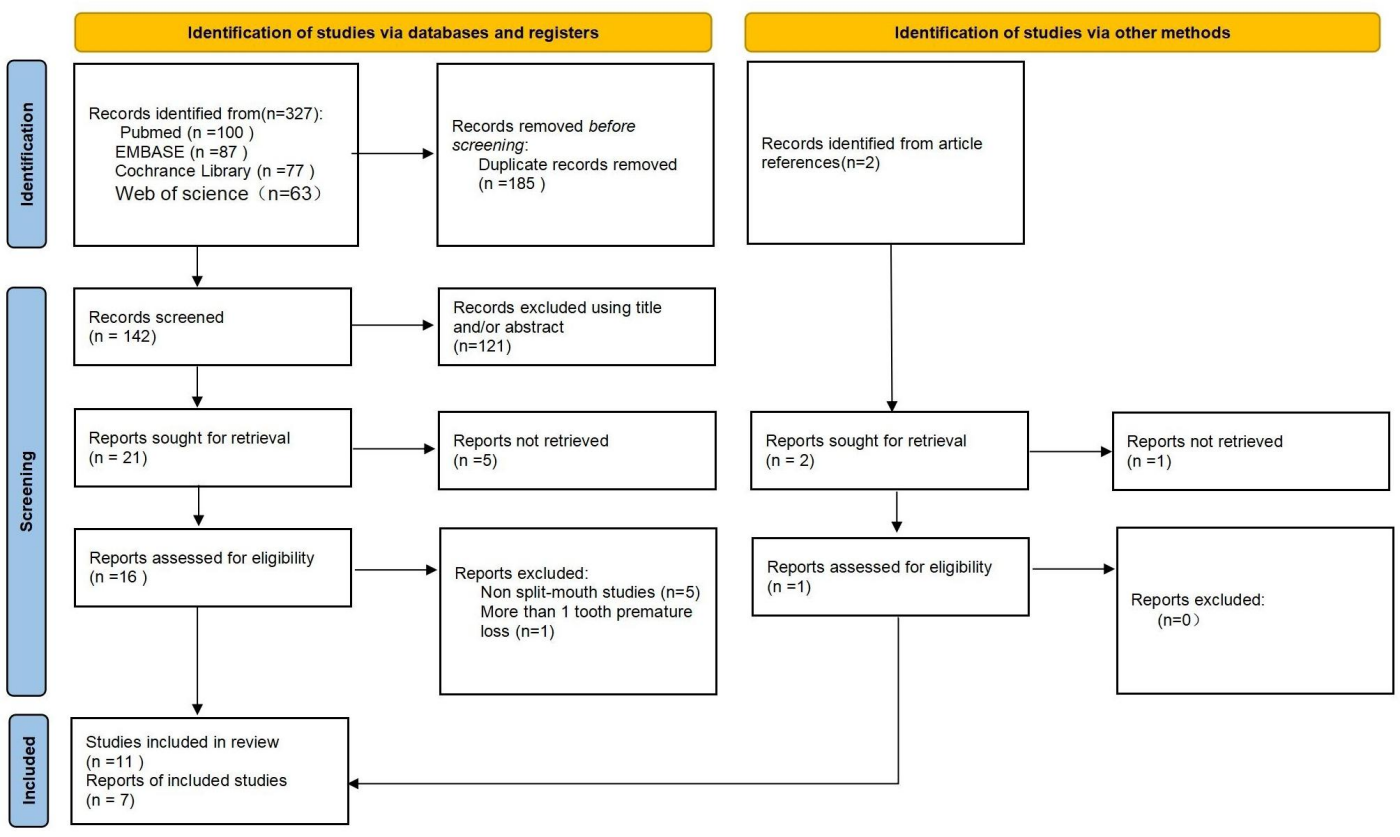
PRISMA flow diagram summarizing the study selection process

## Materials and methods

### Research question and study protocol

This study was registered in the PROSPERO database (Registration number: CRD 42,022,372,202) and conducted in accordance with the Preferred Reporting Items for Systematic Reviews and Meta-Analysis (PRISMA) 2020 guidelines [27]. The research question of this review was based on the PECOS framework: Population (P): children with unilateral first primary molar premature loss in primary dentition and mixed dentition; Exposure (E): unilateral premature loss of a primary first molar; Comparison (C): between side with premature loss of first primary molar and the control side with no loss of molars; Outcome (O): the dental arch spatial changes; and Study Design (S): split-mouth studies were retrieved for analysis.

### Eligibility criteria

Data were included if dental arch spatial changes after unilateral premature loss of first primary molars were investigated in human split-mouth studies. Reviews, abstracts, case reports and series, comments, letters to the editor, conference proceedings, in vitro investigations, and animal studies were excluded.

### Literature search strategy and selection of papers

The PubMed, Cochrane, ClinicalTrials, and EMBASE databases were searched for studies through September 15, 2022. No restrictions were placed on the language and date of publication. Manual searching of references in the relevant published articles was also performed. Three corresponding authors were contacted to either acquire unpublished study results of published trial protocols relevant to our study or clarify information in the original manuscripts. Despite our following up with those authors, none responded to our inquiries. Research publications from the same authors or institutions were scrutinized to eliminate any data redundancy. In the case of redundancy, only results from the most recent publications were included.

The search terms included “primary first molar OR primary maxillary first molar OR primary mandibular first molar,” “tooth migration OR tooth drift OR mesial movement OR distal movement OR space loss OR arch changes,” and “premature tooth loss OR premature loss OR tooth loss OR tooth extraction OR tooth exfoliation.” The search terms were used alone or in combination using the Boolean operators OR and AND (Table 1).

**Table 2:** General characteristics of the studies included in the systematic review and meta-analysis

Author	Study design	Age(years)	Sample size	Arch(n)	Data collection	Follow-up Period	Evaluation indicators	D + E Space loss(mm)
Heidari et al. 2022 [37]	cross-sectional Split-mouth	8–10 (9.08 ± 0.58)	47	Maxilla(25) Mandible(22)	Plaster casts	11.85 ± 5.81 m(6-24 m)	midline, molar and canine relationship, facial growth pattern, Canine’s inclination, space loss, crowding	Maxilla: 0.54 Mandible: 0.58
Mosharrafian et al. 2021 [13]	cross-sectional Split-mouth	6–8 (7.30 ± 0.68)	50	Maxilla(25) Mandible(25)	Plaster casts	13.54 ± 6.28 m (6-24 m)	midline, molar and canine relationship, facial growth pattern, space loss, crowding	Maxilla: 1.32 Mandible: 1.40
Kobylńska et al. 2019 [36]	longitudinal study Split-mouth	5–7 (6.64 ± 1.01)	44*	Maxilla(16) Mandible(14)	Plaster casts	1,3,6,12 m	midline, inter-arch tooth alignment, Angle’s class, vertical bite, lateral teeth contact, radiological assessment, space loss	Maxilla: 1.156 Mandible: 1.000
Lin et al. 2017 [9]	longitudinal study Split-mouth	5–7 (6.0 ± 0.42)	9	Maxilla(9)	Plaster casts	81 m	arch width, arch length, intercanine width, intercanine length, and arch perimeter, space loss	Not mentioned
Alexander et al. 2015 [34]	longitudinal study Split-mouth	7.7–8.2	226	Maxilla(111) Mandible(115)	Direct intraoral measurement	9 m	facial growth pattern, space loss	Maxilla:(Leptoprosopic/End-On:1.75 ± 0.31; Leptoprosopic/Class I:0.89 ± 0.16; Mesoprosopic/Euryprosopic/End-On:+0.07 ± 0.03; Mesoprosopic/Euryprosopic/Class I:+0.11 ± 0.05) Mandible:(Leptoprosopic/End-On:1.38 ± 0.26; Leptoprosopic/Class I:1.71 ± 0.43; Mesoprosopic/Euryprosopic/End-On:1.59 ± 0.43; Mesoprosopic/Euryprosopic/Class I:0.08 ± 0.04)
Lin et al. 2011 [15]	longitudinal study Split-mouth	6–9 (6.0 ± 0.74)	13	Maxilla(13)	Plaster casts	12 m	arch width, arch length, intercanine width, intercanine length, arch perimeter, space loss	Maxilla: 0.82.
Macena et al. 2011 [33]	longitudinal study Split-mouth	6–9	20	Maxilla (12) Mandible (8)	Plaster casts	3、6、10 m	arch length, arch hemi- perimeter, space loss	Maxilla: 0.2^&^ Mandible: 1.0^&^
Park et al. 2009 [32]	cross-sectional Split-mouth	5–10	13	Maxilla(13)	Digitized plaster casts	12 m (8-23 m)	space loss, arch width, arch length, arch perimeter, the inclination and angulation	Maxilla: 0.57 ± 0.83
Kim et al. 2008 [35]	cross-sectional Split-mouth	6–10	6	Maxilla(3) Mandible(3)	Digitized plaster casts	Maxilla: 15.3 m (6-23 m) Mandible: 13.6 m (9-20 m)	space loss, arch width, arch length, arch perimeter, the inclination and angulation	Maxilla: 0.43 Mandible: 1.78
Lin et al. 2007 [31]	longitudinal study Split-mouth	4–7 (5.9 ± 0.74)	19	Maxilla(19)	Plaster casts	6 m	arch width, arch length, intercanine width, intercanine length, and arch perimeter, space loss	Maxilla: 1.08
Padam et al. 2006 [30]	longitudinal study Split-mouth	6–9	40**	Mandible(30)	Plaster casts	2、4、6、8 m	arch length, arch perimeter, arch width, space loss	Mandible: 1.83^&^

*: 17 individuals were excluded from the studies because of malocclusion (n = 4), lack of second premolar buds on examination (n = 1), the presence of mesiodens (n = 2) and further tooth extractions (n = 10)

**:10 individuals were excluded because of no further follow up

^&^ : D space loss

The searches were imported into the EndNote (v. 20) library. Duplications were identified and removed. Titles and abstracts of each retrieved record were screened to exclude any papers not fulfilling inclusion criteria. Two independent reviewers (JZ Zhao and H Jin) evaluated the studies extracted from the searches. When there was uncertainty on the eligibility of an article, the study was adjudicated based on discussion and consensus between the two reviewers and a third reviewer (XR Qin).

### Data extraction

Relevant data from the included papers were independently extracted in duplicate by two authors (JZ Zhao and H Jin). Data extracted from the selected studies included study information (author, year, and study design), patient information (age, sample size, follow-up time, tooth, research methods, and indicators), diagnostic information (facial type, molar occlusal relationship, canine occlusal relationship, crowding, and midline), and follow-up evaluations (D + E and D space: D and E are the first and second primary molar respectively, while D + E space is the space occupied by the first and second primary molars; arch width, length, and perimeter). Because the follow-up times were different in the longitudinal studies, they were divided into three periods, short-term (≤ 6 m), medium-term (6–24 m), and long-term (> 24 m), in order to evaluate results for different follow-up times. Duplication of sample data in meta-analyses was avoided by selecting for analysis only the final data in a longitudinal study.

### Quality assessment

Quality assessment was carried out independently by two authors (JZ Zhao and H Jin), and any disagreements were resolved by consensus. The ROBINS-I (Risk Of Bias In Non-randomized Studies – of Interventions)(http://creativecommons.org/licenses/by-nc-nd/4.0/) tool was used to assess the quality of the studies. The ROBINS-I tool evaluates the methodological quality of individual studies based on seven domains grouped into three phases: (1) Pre-intervention—biases due to confounding and biases in selection of participants into the study; (2) At intervention—biases in classification of interventions; and (3) Post-intervention—biases due to deviations from intended interventions, biases due to missing data, biases in measurement of outcomes, and biases in selection of the reported results. Studies were categorized four levels as critical, serious, moderate, and low respectively.

### Statistical analysis

SPSS 22.0 software (SPSS, Inc., Chicago, IL, USA) was used to apply the Kappa test for assessment of article identification, screening, data extraction, and quality to evaluate agreement among reviewers.

Meta-analysis was performed when warranted by the quality and quantity of data. Review manager 5 software (Revman5.4) was used to analyze the combined effect, heterogeneity, and publication bias. To test for statistical significance in the differences in spaces between the extraction and control sides, and space losses between baseline and final examination values, mean differences (MD) were calculated for D + E and D spaces and space losses, and for arch widths, lengths, and perimeters. Heterogeneity was assessed using I^2^ statistics and Cochrane’s Q test, with I^2^ > 50% or P < 0.10 on Cochrane’s Q test indicating substantial heterogeneity [28]. P-values < 0.05 were considered statistically significant. Publication bias was evaluated by visual inspection of funnel plot [29]. Stata software (Stata 15.1) was used to analyze the results of Begg’s and Egger’s tests and perform sensitivity analyses.

## Results

### Systematic search

The primary search identified 329 published papers, of which 16 satisfied the initial inclusion criteria. Reading of complete texts resulted in the inclusion of 11 studies, 9 longitudinal [9, 15, 30–36] and 2 cross-sectional [13, 37]. Among them, there were five studies on Caucasians [13, 30, 33, 34, 37], five on Mongolians [9, 15, 31, 32, 35], and one on a Central European [36]. Five studies were excluded because they were non split-mouth (n = 5) or nonunilateral premature loss of a primary first molar (n = 1) [1, 4, 14, 26, 38, 39] (Online Resource 1). According to the quality and quantity of data, seven articles were selected for meta-analysis [15, 30, 32, 34–36]. Details on the selection process of research articles are presented in a flow diagram (Fig. 1).

**Fig. 2:**
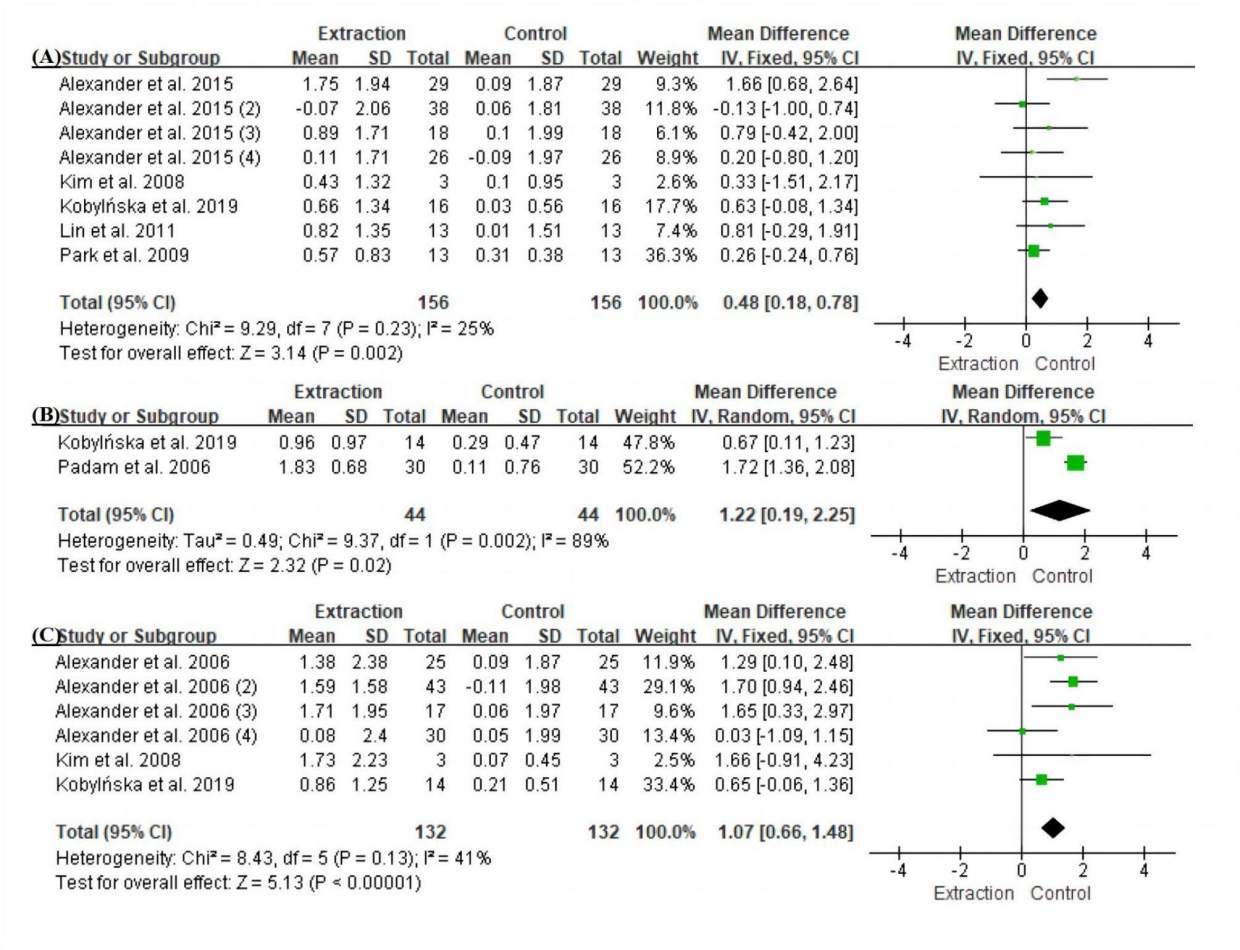
Forest plot of space differences (D/D + E) between the extraction and control sides (A) D + E space differences in the maxilla. (B) D space differences in the mandible. (C) D + E space differences in the mandible. Space was significantly reduced compared with the control side (P < 0.05 each)

**Fig. 3:**
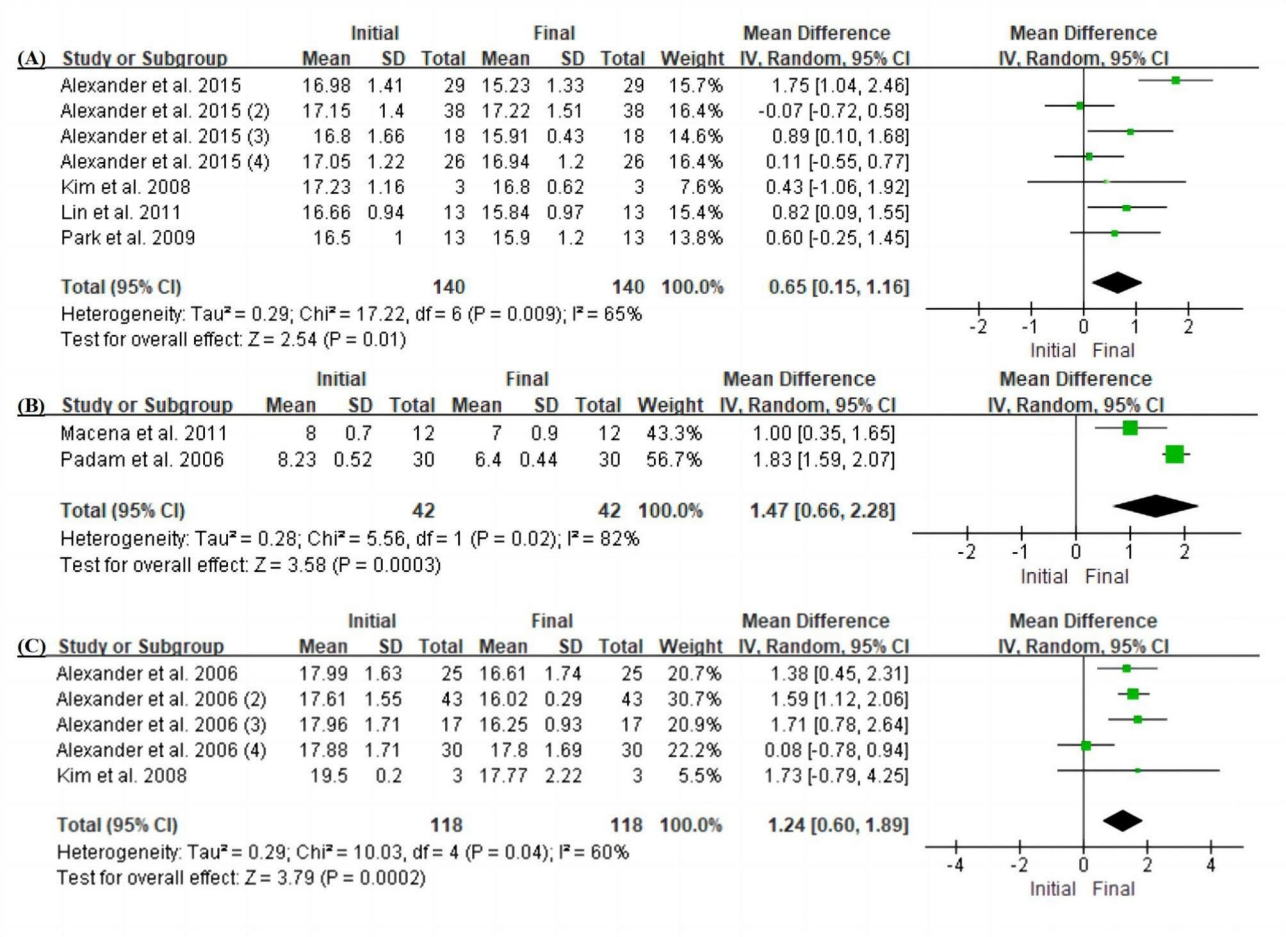
Forest plot of space changes (D/D + E) on the extraction side between the initial baseline values and those at the final follow-up examination. (A) D + E space changes in the maxilla. (B) D space changes in the mandible. (C) D + E space changes in the mandible. Space was significantly reduced compared with the initial baseline values (P < 0.05 each)

**Table 3:** Quality assessment according to ROBINS-I tool of the included observational studies

Study	Pre-intervention		At intervention		Post-intervention			Overall risk of bias
	Due to confounding	Selection of participants into the study	Classification of interventions	Deviations from intended interventions	Missing data	Measurement of outcomes	Selection of the reported result	Low/Moderate/ Serious/Critical
Heidari et al. 2022 [37]	Low	Low	Low	Low	Low	Moderate	Low	Moderate
Mosharrafian et al. 2021 [13]	Low	Low	Low	Low	Low	Moderate	Low	Low
Kobylńska et al. 2019 [36]	Moderate	Low	Low	Low	Low	Moderate	Low	Moderate
Lin et al. 2017 [9]	Moderate	Low	Low	Low	Low	Moderate	Low	Moderate
Alexander et al. 2015 [34]	Low	Low	Low	Low	Low	Moderate	Low	Low
Lin et al. 2011 [15]	Moderate	Low	Low	Low	Low	Moderate	Low	Moderate
Macena et al. 2011 [33]	Moderate	Low	Low	Low	Low	Moderate	Low	Moderate
Park et al. 2009 [32]	Moderate	Low	Low	Low	Low	Low	Low	Moderate
Lin et al. 2007 [31]	Moderate	Low	Low	Low	Low	Moderate	Low	Moderate
Padam et al. 2006 [30]	Moderate	Low	Low	Low	Low	Moderate	Low	Moderate
Kim et al. 2008 [35]	Moderate	Low	Low	Low	Low	Moderate	Low	Moderate

A total of 477 individuals aged 5–10 years were included, comprising 246 cases of premature loss of first primary molars from the maxilla and 217 cases from the mandible. Individuals were excluded from the studies for a variety of reasons (n = 27), including malocclusion (n = 4), lack of second premolar buds on examination (n = 1), the presence of mesiodens (n = 2), further tooth extractions (n = 10), and no further follow up (n = 10) (Table 2).

### Quality assessment and kappa’s test

Quality assessment of the included studies is shown in Table 3. Overall, nine studies were considered to be moderate risk of bias [9, 15, 30–33, 35–37], and two were low risk of bias [13, 34].

The Kappa coefficients of the reviewers involved in article identification and screening, data extraction, and quality assessment were 0.895, 0.892, and 1.000, respectively(Online Resource 2). All were greater than 0.800, indicating strong agreement among reviewers [40].

### Characteristics of the clinical protocol

In general, the children included in these studies were expected to need the unilateral premature extraction of a primary first molar because of caries and/or failed pulp therapy, with an intact contralateral primary first molar available for use as the control. The research data were obtained in three ways: plaster cast [9, 13, 15, 30, 31, 33, 36, 37], digital plaster cast [32, 35], and direct intraoral measurement [34]. The initial study used plaster casts made from alginate impressions just before extraction of the primary first molar [30, 32] or 2–14 days after extraction [9, 15, 31, 36]. Study outcomes were measured before or after tooth loss and at specified time points during the follow-up period. The outcomes were spatial changes between the intervention and control sides as represented by primary molar D + E or D space, and arch width, length, and perimeter. These quantities were measured in all the selected studies except for two that included space loss, midline/molar/canine relationships, facial growth patterns, canine inclination, and crowding [13, 37]. The follow-up time varied from 2 to 81 months [9], with one at 81 months being the extreme, while all others were less than 24 months.

### Short-term (≤ 6 m) space changes

Four articles described short-term follow up (≤ 6 m) [30, 31, 33, 36], including 1–4 and 6 months. However, there were only one or two research articles for each follow-up time, which could not be combined for meta-analysis. However, in these studies, space loss was detected at an early stage. Padma and Retnakumari [30] found the greatest space loss to occur in the 4 months immediately following premature extraction.

### Medium-term (6–24 m) space changes (meta-analysis)

Only two of the included studies assessed first primary molar D space in the maxilla [33, 36], and meta-analysis was not feasible because of incomplete standard deviation data in Kobylińska’s study [36]. In Alexander’s study, the population was divided into four groups according to occlusal relationship and facial type: leptoprosopic with end-on molar occlusions, mesoprosopic/euryprosopic with end-on molar occlusions, leptoprosopic with Class I molar occlusions, and mesoprosopic/euryprosopic with Class I molar occlusions. Each group was included in the meta-analysis as an independent sample. The follow-up period was 6–24 months in the studies included in this meta-analysis.

Over the medium-term follow-up period (6–24 months) [15, 30, 32–36], the D and D + E spaces on the extraction side were significantly smaller than those on the control side in both the maxilla and mandible (D + E space difference in the maxilla: MD 0.48, 95% CI 0.18–0.78, P < 0.01; D space difference in the mandible: MD 1.22, 95% CI 0.19–2.25, P = 0.02; D + E space difference in the mandible: MD 1.07, 95% CI 0.66–1.48, P = 0.02) (Fig. 2A–C). The space loss in the maxillary D + E was 0.65 mm (MD 0.65, 95% CI 0.15–1.16, P = 0.01) (Fig. 3A). The space loss in the mandibular D + E was 1.24 mm (MD 1.24, 95% CI 0.60–1.89, P < 0.01), and that in the mandibular D was 1.47 mm (MD 1.47, 95% CI 0.66–2.28, P < 0.01) (Fig. 3B–C).

**Fig. 4:**
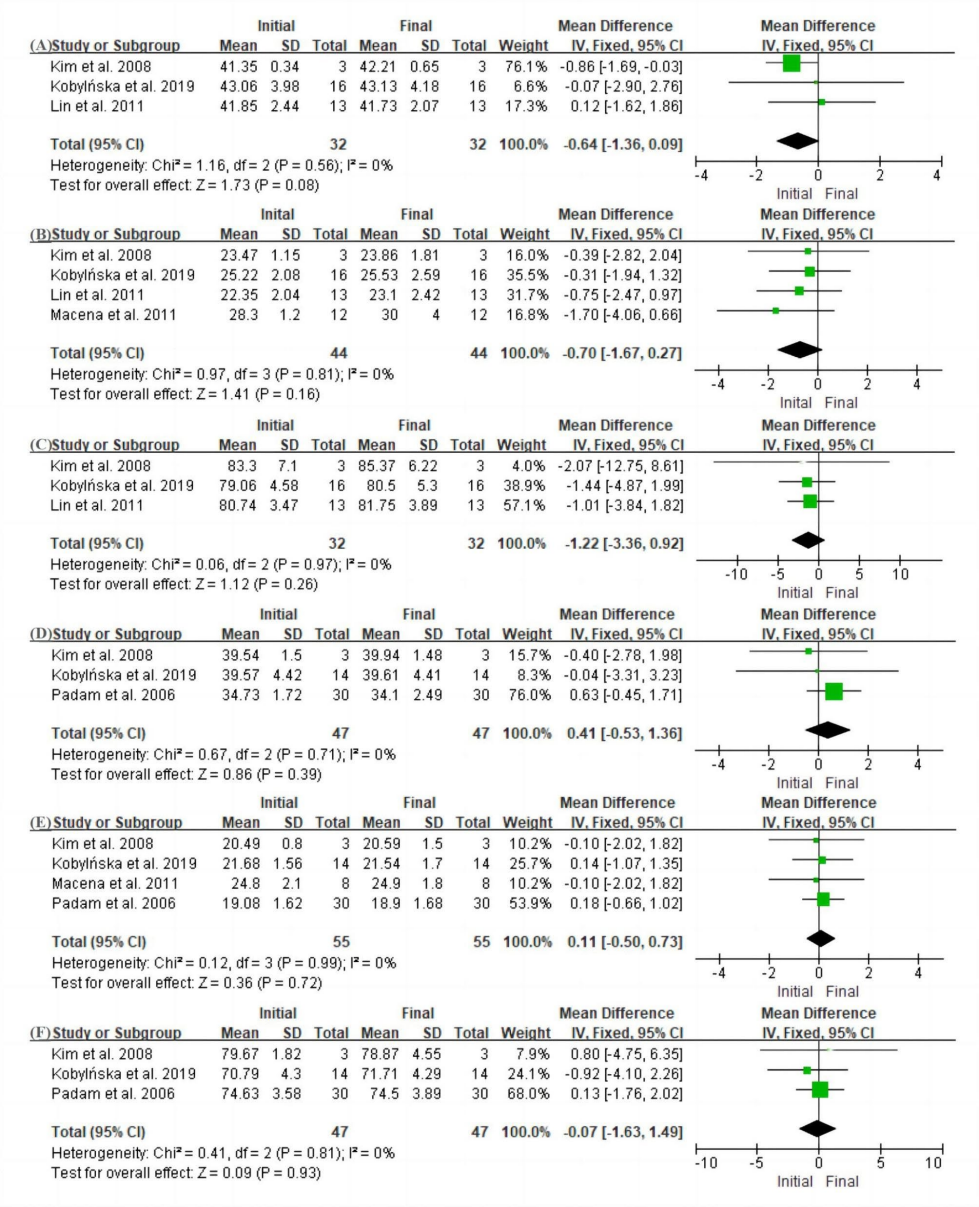
Forest plot of dental arch changes after premature loss of the first primary molar (A) Arch width of the maxilla. (B) Arch length of the maxilla. (C) Arch perimeter of the maxilla. (D) Arch width of the mandible. (E) Arch length of the mandible. (F) Arch perimeter of the mandible. No statistically significant differences were observed between the initial baseline values and those of the final follow-up examinations (P > 0.05 each)

**Fig. 5:**
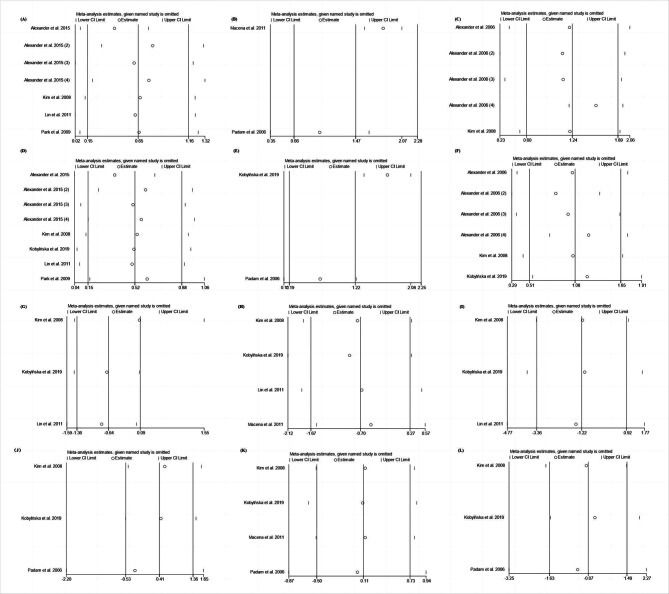
Sensitivity analyses of the included articles (A) D + E space changes in the maxilla on the extraction side. (B) D space changes in the mandible on the extraction side. (C) D + E space changes in the mandible on the extraction side. (D) D + E space differences in the maxilla between the extraction and control sides. (E) D space differences in the mandible between the extraction and control sides. (F) D + E space differences in the mandible between the extraction and control sides. (G) Arch width of the maxilla. (H) Arch length of the maxilla. (I) Arch perimeter of the maxilla. (J) Arch width of the mandible. (K) Arch length of the mandible. (L) Arch perimeter of the mandible. Sensitivity analysis showed that, except for the meta-analysis of D space and D loss, the combined effects did not change after excluding any single study, suggesting that the results were generally reliable

**Fig. 6:**
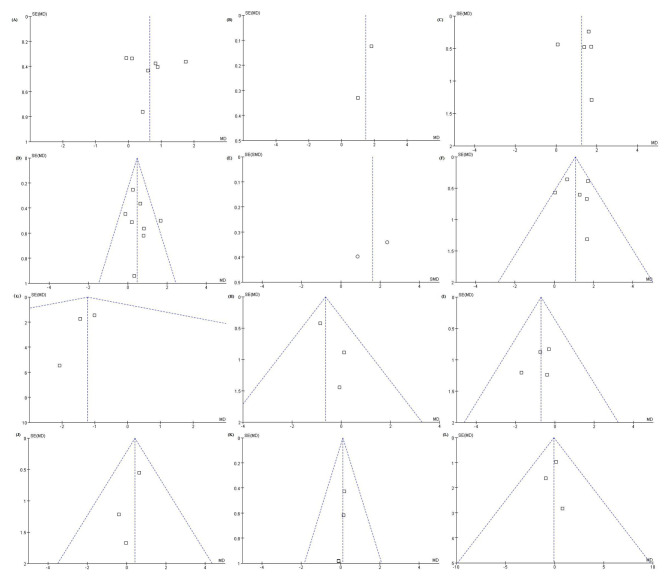
Funnel plot of the included articles (A) D + E space changes in the maxilla on the extraction side. (B) D space changes in the mandible on the extraction side. (C) D + E space changes in the mandible on the extraction side. (D) D + E space differences in the maxilla between the extraction and control sides. (E) D space differences in the mandible between extraction and control sides. (F) D + E space differences in the mandible between the extraction and control sides. (G) Arch width of the maxilla. (H) Arch length of the maxilla. (I) Arch perimeter of the maxilla. (J) Arch width of the mandible. (K) Arch length of the mandible. (L) Arch perimeter of the mandible. The funnel plots indicated that there was no obvious heterogeneity among the included studies

**Table 4:** Begg’s and Egger’s tests of D and D + E space changes and dental arch changes

	Begg’s test		Egger’s test	
	Z	P	t	P
D + E space of Maxilla on extraction side	0.30	0.764	-0.21	0.845
D space of Mandible on extraction side	0.00	1.000	-*	-*
D + E space of Mandible on extraction side	0.24	0.806	-0.19	0.861
D + E space changes of Maxilla between extraction and control side	0.62	0.536	-0.80	0.457
D space changes of Mandible between extraction and control side	0.00	1.000	-*	-*
D + E space changes of Mandible between extraction and control side	0.38	0.707	-0.25	0.816
Arch width of Maxilla	0.00	1.000	-1.68	0.341
Arch width of Mandible	0.00	1.000	1.80	0.322
Arch length of Maxilla	0.34	0.734	0.86	0.430
Arch length of Mandible	1.02	0.308	3.00	0.096
Arch perimeter of Maxilla	0.00	1.000	1.23	0.434
Arch perimeter of Mandible	0.00	1.000	0.02	0.985

* There were only two articles included, so the Egger’s test was not performed

Five studies assessed dental arch changes [15, 30, 33, 35, 36]. However, no significant differences were found in arch width, arch length, or arch perimeter between the initial examination and the medium-term follow-up examination ( P > 0.05) (Fig. 4A–F). In addition, there were three articles from one institution evaluating both intercanine width and length [9, 15, 31] in which the authors found that both were significantly larger at the 6, 12, and 81 month follow-ups.

Sensitivity analysis showed that the combined effect did not change after excluding any of the studies, suggesting that the results were reliable, except for the meta-analysis of D space and loss (Fig. 5). Only two studies were included, and the results could not be obtained by removing the study. Therefore, further research is needed to verify these results. Funnel plots, Begg’s tests, and Egger’s tests showed that there was no publication bias in the included studies (Fig. 6; Table 4, Online Resource 3 and 4).

### Long-term (> 24 m) space changes

Lin et al. [9] evaluated unilateral premature loss of the primary maxillary first molar in nine children (6.0 ± 0.42 years old), involving a follow-up period of 81 months, which was the longest followup period in all the included studies. The arch width, arch length, and intercanine width and length significantly increased over that period.

### Factors influencing space change after premature loss

There were two studies [13, 37] of multiple factors affecting space change after the first primary molar loss, including age (years); tooth extraction time (months); molar relationships on the control side; facial patterns; and canine movements, crowding, and relationships on the control side, midline and jaw. Factors such as age, facial pattern, duration of tooth loss, molar relationships on the control side, and canine-to-lateral distance influenced space loss following premature loss of the first primary molars [13, 37]. However, other factors such as crowding, midline deviation, and canine relationships on the control side did not significantly affect space loss [37].

## Discussion

The present systematic review and meta-analysis identified studies that evaluated space changes following premature loss of the primary first molar. Because systematic reviews are based on rigorous inclusion, exclusion, and methodological criteria, few articles addressing this topic are available, with most lacking a self-controlled group [1, 4]. Additionally, in some articles, there is more than one missing molar in a single jaw [4, 26, 41, 42], which may be another reason for relatively limited research in this area.

Nine studies were considered to be moderate risk of bias [9, 15, 30–33, 35–37], and two were low risk of bias [13, 34]. In two articles [30, 36], 27 individuals were excluded because of malocclusion, lack of second premolar buds on examination, the presence of mesiodens, additional tooth extractions, or no further follow up, which may not be serious sources of bias for the study outcomes. However, except for one study [9] that was followed up for 81 months, all included studies were followed up for less than 24 months, which may reduce the validity of the outcomes.

Over the medium-term follow-up period (6–24 months), the D or D + E space on the extraction side was significantly smaller than on the control side in both the maxilla and mandible (D + E space difference in the maxilla: MD 0.48, 95% CI 0.18–0.78, P < 0.01; D space difference in the mandible: MD 1.22, 95% CI 0.19–2.25, P = 0.02; D + E space difference in the mandible: MD 1.07, 95% CI 0.66–1.48, P = 0.02), which means that if the tooth loss lasts for more than 6 months, the space will be significantly reduced. The space loss in the maxillary D + E was 0.65 mm (MD 0.65, 95% CI 0.15–1.16, P = 0.01), similar to that in Heidari’s study (0.54 mm) [37] but different from that in Tunison’s study (≤ 1 mm) [43]. The space loss in the mandibular D + E was 1.24 mm (MD 1.24, 95% CI 0.60–1.89, P < 0.01), and that in the mandibular D was 1.47 mm (MD 1.47, 95% CI 0.66–2.28, P < 0.01), which was similar to the values reported by Andreeva et al. (1.12–1.50 mm) [26, 43, 44].

In all the included studies, seven articles mentioned the movement of adjacent teeth on the extraction side [15, 30–32, 34–36]. Kobylńska et al. [36] found that the loss of space was due to distalization of the primary canine and mesialization of the second primary molar on both the maxillary and mandibular arches. In the maxillary arch, the space changes consisted mainly of distal drift of the primary canine, but there was no observation of mesial movement of permanent molars or tilting of the primary molars [15, 31]. In the mandibular arch, both mesial migration of posterior teeth and distal movement of anterior teeth were observed, but the distal movement of the primary canine toward the extraction space was most likely responsible for the early space change [30]. In Park and Kim’s studies [32, 35], inclination and angulation of adjacent teeth were measured by 3D scanning and superimposing the initial and final dental casts, but there were no consistent findings concerning inclination and angulation changes on the extraction side in their studies.

According to the seven articles included, the factors influencing the movement of the adjacent teeth may be related to the dental arch, the eruptive status of the first permanent molar, and the occlusal relationship of the molar and facial types [15, 30–32, 34–36]. Alexander et al. [34] analyzed the pattern of space loss in people with different facial types and molar occlusal relationships and observed different movements of adjacent teeth. In people with leptoprosopic facial forms, maxillary space loss occurred by mesial migration of distal segments in all subjects; in the mandibular arch, space loss in more than 80% of subjects occurred by mesial migration of distal segments and distal tipping of the canine, and less than 20% occurred only by mesial migration. In people with end-on molar occlusions and mesoprosopic/euryprosopic facial forms, space loss occurred by mesial migration of distal segments and distal tipping of the canine in 88.37% of subjects, and by mesial migration in only 11.63% of subjects in the mandibular extraction group. No significant differences were found in arch width, arch length, or arch perimeter between the initial baseline values and those after the medium-term follow-up period (6–24 months) (P > 0.05) [15, 30, 33, 35, 36], which may suggest that premature loss of a first primary molar will not affect the development of the dental arch. In addition, the authors found an increase in length and width of the intercanine arch at the 6, 12, and 81 month follow-ups [9, 15, 31], which may provide enough space for eruption of the successor permanent teeth, partially compensating for earlier space loss.

In these split-mouth longitudinal studies, space loss was detected at an early follow-up time (≤ 6 mm) [30, 31, 33, 36]. Padma and Retnakumari [30] observed the greatest space loss in the first 4 months after premature extraction, but space loss subsequently increased gradually and became stable over 6–24 months [30, 33, 34, 36]. Lin and Chang found more than 1 mm space loss in the maxilla at 6 and 12 months, however, and at 81 months follow up, 88.9% of the subjects did not show crowded permanent successors or canine block-out at the extraction site, which suggested that space maintainers were not needed for children aged about 6 years when the permanent first molars were about to erupt or had just erupted. In Heidari’s study [37], space loss resulting from extraction of the first primary molars in late mixed dentition at 8–10 years old was neither statistically nor clinically significant. In addition, some other factors influenced space change after premature loss of the first primary molar. In these studies using multifactor linear regression [13, 37], it was found that factors such as age, facial pattern, duration of tooth loss, and molar relationships influenced space loss following premature loss of the first primary molars. However, two articles are insufficient to confirm this conclusion, and further multifactorial research is needed.

The present study had several limitations. First, the meta-analysis of D space and loss thereof included only two articles, which cannot be improved by removing one article to improve the reliability of the results. Second, follow-up times differed among the included studies, with most having been less than 24 months, so it may be necessary to extend the follow-up times until eruption of the successor permanent teeth occurs.

## Conclusions

Over the medium-term follow-up period (6–24 months), space loss was 0.65 mm for the maxillary D + E, 1.24 mm for the mandibular D + E, and 1.47 mm for the mandibular D. After premature loss of first primary molars, space can be lost, but the amount of loss would not affect arch width, length, or arch perimeter over the 6–24 months follow-up period Factors such as age, time since tooth extraction, facial pattern, and molar relationships also influenced the space change after the premature loss of the first primary molar. It is advisable to precisely assess these related factors to decide whether to place a space maintainer for a prematurely lost primary first molar.

## Electronic supplementary material

Below is the link to the electronic supplementary material.

Supplementary Material 1: Table S1. List of excluded studies with the reasons for exclusion (n = 6).

Supplementary Material 2: Table S2. Interexaminer and intraexaminer Kappa values for article identification and screening, data extraction, and quality assessment.

Supplementary Material 3: Figure S1. Begg’s test of space changes (D/D + E) and dental arch changes after premature loss of the first primary molar. (A) D + E space changes in the maxilla on the extraction side. (A) D + E space changes in the maxilla on the extraction side. (B) D space changes in the mandible on the extraction side. (C) D + E space changes in the mandible on the extraction side. (D) D + E space differences in the maxilla between the extraction and control sides. (E) D space differences in the mandible between the extraction and control sides. (F) D + E space differences in the mandible between the extraction and control sides. (G) Arch width of the maxilla. (H) Arch length of the maxilla. (I) Arch perimeter of the maxilla. (J) Arch width of the mandible. (K) Arch length of the mandible. (L) Arch perimeter of the mandible. Begg’s test indicated that there was no obvious heterogeneity among the included studies.

Supplementary Material 4: Figure S2. Egger’s test of space changes (D/D + E) and dental arch changes after premature loss of the first primary molar. (A) D + E space changes in the maxilla on the extraction side. (B) D space changes in the mandible on the extraction side. (C) D + E space changes in the mandible on the extraction side. (D) D + E space differences in the maxilla between the extraction and control sides. (E) D space differences in the mandible between the extraction and control sides. (F) D + E space differences in the mandible between the extraction and control sides. (G) Arch width in the maxilla. (H) Arch length of the maxilla. (I) Arch perimeter of the maxilla. (J) Arch width of the mandible. (K) Arch length of the mandible. (L) Arch perimeter of the mandible. Egger’s test indicated that there was no obvious heterogeneity among the included studies.

Supplementary Material 5: The PRISMA checklist of this systematic review and meta-analysis

## Data Availability

The datasets used and/or analyzed during the current study are available from the corresponding author on reasonable request.

## Abbreviations

ROBINS-I: Risk Of Bias In Non-randomized Studies – of Interventions
MD: Mean differences.
