# Supplementary material for: Dental arch spatial changes after premature loss of first primary molars: a systematic review and meta-analysis of split-mouth studies
Source: BMC Oral Health. 2023 Jun 28;23:430. doi: 10.1186/s12903-023-03111-x (PMC10304618; doi:10.1186/s12903-023-03111-x)
Supplement: Supplementary file 1 — Supplementary Material 1: Table S1. List of excluded studies with the reasons for exclusion (n = 6). [file 12903_2023_3111_MOESM1_ESM.doc]

Table S1 List of excluded studies with the reasons for exclusion (n = 6).

| Author/year | Title | Reason for exclusion |
| --- | --- | --- |
| Johnsen. 1980[14] | Space observation following loss of the mandibular first primary molars in mixed dentition | Non unilateral premature loss of a primary maxillary first molar |
| Northway et al. 1984[1] | Effects of premature loss of deciduous molars | Non split-mouth study |
| Cernei et al. 2015[38] | The influence of premature loss of temporary upper molars on permanent molars | Non split-mouth study |
| Radosveta et al. 2016[26] | Loss of space according to the time and the type of the premature extracted deciduous teeth | Non split-mouth study |
| P.B.R et al. 2020[39] | Assessment of dental arch space maintenance after the early extraction of the baby tooth | Non split-mouth study |
| Northway. 2000[4] | The not-so-harmless maxillary primary first molar extraction | Non split-mouth study |
