# Supplementary material for: Dental arch spatial changes after premature loss of first primary molars: a systematic review and meta-analysis of split-mouth studies
Source: BMC Oral Health. 2023 Jun 28;23:430. doi: 10.1186/s12903-023-03111-x (PMC10304618; doi:10.1186/s12903-023-03111-x)
Supplement: Supplementary file 2 — Supplementary Material 2: Table S2. Interexaminer and intraexaminer Kappa values for article identification and screening, data extraction, and quality assessment. [file 12903_2023_3111_MOESM2_ESM.doc]

Table S2 Interexaminer and intraexaminer Kappa values for article identification and screening, data extraction, and quality assessment

|  | Kappa | *P* value |
| --- | --- | --- |
| article identification and screening (JZ Zhao and H Jin) | 0.895 | .0001 |
| data extraction (JZ Zhao and H Jin) | 0.892 | .0001 |
| quality assessment (JZ Zhao and H Jin) | 1.000 | .0001 |
