# Supplementary material for: Dental arch spatial changes after premature loss of first primary molars: a systematic review and meta-analysis of split-mouth studies
Source: BMC Oral Health. 2023 Jun 28;23:430. doi: 10.1186/s12903-023-03111-x (PMC10304618; doi:10.1186/s12903-023-03111-x)
Supplement: Supplementary file 3 — Supplementary Material 3: Figure S1. Begg’s test of space changes (D/D + E) and dental arch changes after premature loss of the first primary molar. (A) D + E space changes in the maxilla on the extraction side. (A) D + E space changes in the maxilla on the extraction side. (B) D space changes in the mandible on the extraction side. (C) D + E space changes in the mandible on the extraction side. (D) D + E space differences in the maxilla between the extraction and control sides. (E) D space differences in the mandible between the extraction and control sides. (F) D + E space differences in the mandible between the extraction and control sides. (G) Arch width of the maxilla. (H) Arch length of the maxilla. (I) Arch perimeter of the maxilla. (J) Arch width of the mandible. (K) Arch length of the mandible. (L) Arch perimeter of the mandible. Begg’s test indicated that there was no obvious heterogeneity among the included studies. [file 12903_2023_3111_MOESM3_ESM.doc]

**Supplementary files**


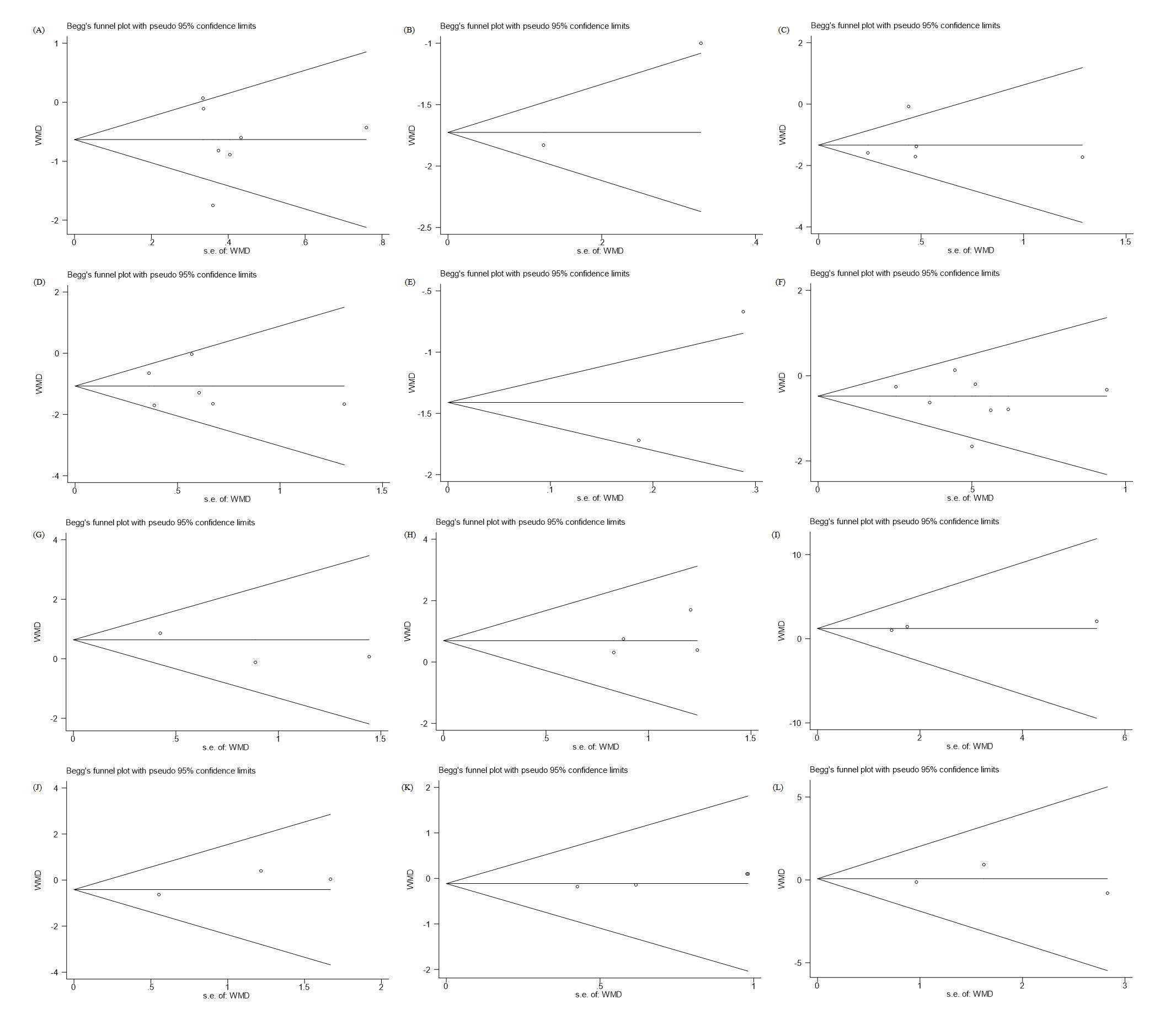


**Fig. S1**  Begg’s test of space changes (D/D+E) and dental arch changes after premature loss of the first primary molar. (A) D+E space changes in the maxilla on the extraction side. (A) D+E space changes in the maxilla on the extraction side. (B) D space changes in the mandible on the extraction side. (C) D+E space changes in the mandible on the extraction side. (D) D+E space differences in the maxilla between the extraction and control sides. (E) D space differences in the mandible between the extraction and control sides. (F) D+E space differences in the mandible between the extraction and control sides. (G) Arch width of the maxilla. (H) Arch length of the maxilla. (I) Arch perimeter of the maxilla. (J) Arch width of the mandible. (K) Arch length of the mandible. (L) Arch perimeter of the mandible. Begg’s test indicated that there was no obvious heterogeneity among the included studies.
