# Supplementary material for: Dental arch spatial changes after premature loss of first primary molars: a systematic review and meta-analysis of split-mouth studies
Source: BMC Oral Health. 2023 Jun 28;23:430. doi: 10.1186/s12903-023-03111-x (PMC10304618; doi:10.1186/s12903-023-03111-x)
Supplement: Supplementary file 5 — Supplementary Material 5: The PRISMA checklist of this systematic review and meta-analysis [file 12903_2023_3111_MOESM5_ESM.docx]

**The PRISMA checklist of this systematic review and meta-analysis**

| **Section and Topic** | **Item #** | **Checklist item** | **Location where item is reported** |
| --- | --- | --- | --- |
| **TITLE** | | |  |
| Title | 1 | Identify the report as a systematic review. | Title （Page 1, Line 1-1)(Page 2, Line 17-18)  Dental arch spatial changes after premature loss of first primary molars: A systematic review and meta-analysis of split-mouth studies |
| **ABSTRACT** | | |  |
| Abstract | 2 | See the PRISMA 2020 for Abstracts checklist.  Provide a structured summary including, as applicable: background; objectives; data sources; study eligibility criteria participants, and interventions; study appraisal and synthesis methods; results; limitations; conclusions and implications of key findings, systematic review registration number. | Abstract( Page 2)  **Background and objectives**: Line 21-22  Data sources: Line 22-23  **Study eligibility criteria participants and interventions:** Line 23-25  **Study appraisal and synthesis methods:** Line25-27  **Results**: Line 27-36  **Conclusions and implications of key findings:** Line 37-39  The **limitations** is described in DISCUSSION, not described in abstract.( Page 12 Line 323-327).  **Registration** number：CRD 42022372202（ Page 4 Line 89） |
| **INTRODUCTION** | | |  |
| Rationale | 3 | Describe the rationale for the review in the context of existing knowledge. | **Background(**Page 2-4)  Introduce the disadvantages of premature loss of first primary molars : Line 44-71  Introduce the debate for space maintenance: Line 72-84. |
| Objectives | 4 | Provide an explicit statement of the objective(s) or question(s) the review addresses. | **Background**(Page 4)  Line 84-86 |
| **METHODS** | | |  |
| Eligibility criteria | 5 | Specify the inclusion and exclusion criteria for the review and how studies were grouped for the syntheses. | Page 4 Line 99-102 |
| Information sources | 6 | Specify all databases, registers, websites, organisations, reference lists and other sources searched or consulted to identify studies. Specify the date when each source was last searched or consulted. | Data sources**.**(Page 4 Line 104-105 )  **Data sources:**PubMed, Cochrane, ClinicalTrials and EMBASE databases  **Date last searched:** September 15, 2022 |
| Search strategy | 7 | Present the full search strategies for all databases, registers and websites, including any filters and limits used. | **Table 1 Search strategy** |
| Selection process | 8 | Specify the methods used to decide whether a study met the inclusion criteria of the review, including how many reviewers screened each record and each report retrieved, whether they worked independently, and if applicable, details of automation tools used in the process. | Study Selection (Page 5 Line 118-123)  Fig. 1 PRISMA flow diagram summarizing the study selection process.  The searches were imported into the EndNote version 20 library. Duplications were identified and removed. (Line 118-119)  Titles and abstracts of each retrieved record were screened to exclude any papers not fulfilling inclusion criteria. Two independent reviewers (JZ Zhao and H Jin) evaluated the studies extracted from the searches. When there was uncertainty on the eligibility of an article, the study was adjudicated based on discussion and consensus between the two reviewers and a third reviewer (XR Qin).( Line 119-123) |
| Data collection process | 9 | Specify the methods used to collect data from reports, including how many reviewers collected data from each report, whether they worked independently, any processes for obtaining or confirming data from study investigators, and if applicable, details of automation tools used in the process. | (Page 5 Line 124-126) |
| Data items | 10a | List and define all outcomes for which data were sought. Specify whether all results that were compatible with each outcome domain in each study were sought (e.g. for all measures, time points, analyses), and if not, the methods used to decide which results to collect. | Data extraction (Page 5 ).  outcomes**:** D+E and D space; arch width, length, and perimeter(Page 5 Line 129-130)  Table 2 General characteristics of the studies included in the systematic review and meta-analysis. |
|  | 10b | List and define all other variables for which data were sought (e.g. participant and intervention characteristics, funding sources). Describe any assumptions made about any missing or unclear information. | Data extraction (Page 5 ).  **Other Variables:** study information (author, year, and study design), patient information (age, sample size, follow-up time, tooth, research methods, and indicators), diagnostic information (facial type, molar occlusal relationship, canine occlusal relationship, crowding, and midline) ( Page 5 Line 126-134)  Three corresponding authors were contacted to either acquire unpublished study results of published trial protocols relevant to our study or clarify information in the original manuscripts. (Page 4 Line 107-110)  Table 2 Patients, teeth, pretreatment signs and symptoms, and treatment information of the included studies. |
| Study risk of bias assessment | 11 | Specify the methods used to assess risk of bias in the included studies, including details of the tool(s) used, how many reviewers assessed each study and whether they worked independently, and if applicable, details of automation tools used in the process. | Page 5 Line 136-145. |
| Effect measures | 12 | Specify for each outcome the effect measure(s) (e.g. risk ratio, mean difference) used in the synthesis or presentation of results. | Mean differences (MD) were calculated for D+E and D spaces and space losses, and for arch widths, lengths, and perimeters.( Page 6 Line 153-155) |
| Synthesis methods | 13a | Describe the processes used to decide which studies were eligible for each synthesis (e.g. tabulating the study intervention characteristics and comparing against the planned groups for each synthesis (item #5)). | Because the follow-up times were different in the longitudinal studies, they were divided into three periods, short-term (≤6 m), medium-term (6–24 m), and long-term (>24 m), in order to evaluate results for different follow-up times. Duplication of sample data in meta-analyses was avoided by selecting for analysis only the final data in a longitudinal study.( Page 5 Line 130-134) |
|  | 13b | Describe any methods required to prepare the data for presentation or synthesis, such as handling of missing summary statistics, or data conversions. | Three corresponding authors were contacted to either acquire unpublished study results of published trial protocols relevant to our study or clarify information in the original manuscripts. (Page 4 Line 107-109) |
|  | 13c | Describe any methods used to tabulate or visually display results of individual studies and syntheses. | (Page 6 Line 150-160) |
|  | 13d | Describe any methods used to synthesize results and provide a rationale for the choice(s). If meta-analysis was performed, describe the model(s), method(s) to identify the presence and extent of statistical heterogeneity, and software package(s) used. | Review manager 5 software (Revman5.4) was used to analyze the combined effect, heterogeneity, and publication bias. Heterogeneity was assessed using I^2^ statistics and Cochrane’s Q test, with I^2^ > 50% or P < 0.10 on Cochrane’s Q test indicating substantial heterogeneity. P-values <0.05 were considered statistically significant. Publication bias was evaluated by visual inspection of funnel plot(Page 6 Line 151-158). |
|  | 13e | Describe any methods used to explore possible causes of heterogeneity among study results (e.g. subgroup analysis, meta-regression). | Stata software (Stata 15.1) was used to analyze the results of Begg’s and Egger’s tests and perform sensitivity analyses.(Page 6 Line 158-160) |
|  | 13f | Describe any sensitivity analyses conducted to assess robustness of the synthesized results. | **Sensitivity analyses:** Stata software (Stata 15.1) was used to analyze the results of Begg’s and Egger’s tests and perform sensitivity analyses.(Page 6 Line 158-160) |
| Reporting bias assessment | 14 | Describe any methods used to assess risk of bias due to missing results in a synthesis (arising from reporting biases). | The potential for publication bias was assessed using funnel plot, Bgger’s and Egg’s test.(Page 6 Line 158-160) |
| Certainty assessment | 15 | Describe any methods used to assess certainty (or confidence) in the body of evidence for an outcome. | SPSS 22.0 software (SPSS, Inc., Chicago, IL, USA) was used to apply the Kappa test for assessment of article identification, screening, data extraction, and quality to evaluate agreement among reviewers.(Page 6 Line 147-149) |
| **RESULTS** | | |  |
| Study selection | 16a | Describe the results of the search and selection process, from the number of records identified in the search to the number of studies included in the review, ideally using a flow diagram. | Page 6-7 Line 163-171  Fig. 1 PRISMA flow diagram summarizing the study selection process. |
|  | 16b | Cite studies that might appear to meet the inclusion criteria, but which were excluded, and explain why they were excluded. | Reasons for exclusions: Five studies were excluded because they were non split-mouth (n=5) or nonunilateral premature loss of a primary first molar (n=1) (Additional file 1).(Page 7 Line 167-169)  Additional file 1 List of excluded studies with the reasons for exclusion (n = 6). |
| Study characteristics | 17 | Cite each included study and present its characteristics. | Search Results(Page 6 Line 163-165)  Table 2. Table 2 General characteristics of the studies included in the systematic review and meta-analysis |
| Risk of bias in studies | 18 | Present assessments of risk of bias for each included study. | Quality assessment and Kappa’s test (Page 7 Line 178-183)  Table 3 Quality assessment according to Newcastle Ottawa scale of the included observational studies |
| Results of individual studies | 19 | For all outcomes, present, for each study: (a) summary statistics for each group (where appropriate) and (b) an effect estimate and its precision (e.g. confidence/credible interval), ideally using structured tables or plots. | Characteristics of the clinical protocol(Page 7-8 Line 185-198)  Medium-term (6–24 m) space changes (meta-analysis) (Page 7 Line 206-2228).  Fig. 2 Forest plot of space differences (D/D+E) between the extraction and control sides.  Fig. 3 Forest plot of space changes (D/D+E) on the extraction side between the initial baseline values and those at the final follow-up examination.  Fig. 4 Forest plot of dental arch changes after premature loss of the first primary molar |
| Results of syntheses | 20a | For each synthesis, briefly summarise the characteristics and risk of bias among contributing studies. | Funnel plots and Begg tests and Egg tests showed that there were no publication bias in the included literature(Fig. 6, Table 4, Additional files 3, 4).（Page 9 Line 229-234）  Fig. 6 Funnel plot of the included articles.  Table 4 Begg’s and Egger’s tests of D and D+E space changes and dental arch changes  Additional file 3 Begg’s test of space changes (D/D+E) and dental arch changes after premature loss of the first primary molar.  Additional file 4. Egger’s test of space changes (D/D+E) and dental arch changes after premature loss of the first primary molar. |
|  | 20b | Present results of all statistical syntheses conducted. If meta-analysis was done, present for each the summary estimate and its precision (e.g. confidence/credible interval) and measures of statistical heterogeneity. If comparing groups, describe the direction of the effect. | **Medium-term (6–24 m) space changes (meta-analysis)** (Page 7 Line 206-2228).  Fig. 2 Forest plot of space differences (D/D+E) between the extraction and control sides.  Fig. 3 Forest plot of space changes (D/D+E) on the extraction side between the initial baseline values and those at the final follow-up examination.  Fig. 4 Forest plot of dental arch changes after premature loss of the first primary molar. |
|  | 20c | Present results of all investigations of possible causes of heterogeneity among study results. | (Page10 Line 278-286) |
|  | 20d | Present results of all sensitivity analyses conducted to assess the robustness of the synthesized results. | Sensitivity analysis showed that the combined effect did not change after excluding any of the studies, suggesting that the results were reliable, except for the meta-analysis of D space and loss (Fig. 5). Only two studies were included, and the results could not be obtained by removing the study. .(Page 9 Line 231-232)  Fig. 5 Sensitivity analyses of the included articles. |
| Reporting biases | 21 | Present assessments of risk of bias due to missing results (arising from reporting biases) for each synthesis assessed. | Funnel plots and Begg tests and Egg tests showed that there were no publication bias in the included literature(Fig. 6, Table 4, Additional files 3, 4).（Page 9 Line 232-234）  Fig. 6 Funnel plot of the included articles.  Table 4 Begg’s and Egger’s tests of D and D+E space changes and dental arch changes  Additional file 3 Begg’s test of space changes (D/D+E) and dental arch changes after premature loss of the first primary molar.  Additional file 4. Egger’s test of space changes (D/D+E) and dental arch changes after premature loss of the first primary molar. |
| Certainty of evidence | 22 | Present assessments of certainty (or confidence) in the body of evidence for each outcome assessed. | **Measures of consistency:** The Kappa coefficients of the reviewers involved in article identification and screening, data extraction, and quality assessment were 0.895, 0.892, and 1.000, respectively (Additional file 2). All were greater than 0.800, indicating strong agreement among reviewers.(Page 7 Line 181-183) |
| **DISCUSSION** | | |  |
| Discussion | 23a | Provide a general interpretation of the results in the context of other evidence. | **Discussion** (Page 10-12 Line 271-327) |
|  | 23b | Discuss any limitations of the evidence included in the review. | The present study had several limitations. First, the meta-analysis of D space and loss thereof included only two articles, which cannot be improved by removing one article to improve the reliability of the results. Second, follow-up times differed among the included studies, with most having been less than 24 months, so it may be necessary to extend the follow-up times until eruption of the successor permanent teeth occurs.(Page 12 Line 323-327)  However, except for one study[9] that was followed up for 81 months, all included studies were followed up for less than 24 months, which may reduce the validity of the outcomes. (Page 10 Line 284-286) |
|  | 23c | Discuss any limitations of the review processes used. | However, two articles are insufficient to confirm this conclusion, and further multifactorial research is needed. (Page 12 Line321-322) |
|  | 23d | Discuss implications of the results for practice, policy, and future research. | **Conclusion**(Page 12 Line 329-326) |
| **OTHER INFORMATION** | | |  |
| Registration and protocol | 24a | Provide registration information for the review, including register name and registration number, or state that the review was not registered. | This study was registered in the PROSPERO database (Registration number: CRD 42022372202) and conducted in accordance with the Preferred Reporting Items for Systematic Reviews and Meta-Analysis (PRISMA) 2020 guidelines(Page 4 Line 89-97) |
|  | 24b | Indicate where the review protocol can be accessed, or state that a protocol was not prepared. | The protocol is described in the Methods. A protocol was not prepared. |
|  | 24c | Describe and explain any amendments to information provided at registration or in the protocol. | The protocol is described in the Methods and Registration (CRD 42022372202). |
| Support | 25 | Describe sources of financial or non-financial support for the review, and the role of the funders or sponsors in the review. | **Funding**: Supported by Assistant Research Fund for the president of Jinan Stomatological Hospital (2019-05) . |
| Competing interests | 26 | Declare any competing interests of review authors. | The authors declare that they have no competing interests.(Page 13 Line 355) |
| Availability of data, code and other materials | 27 | Report which of the following are publicly available and where they can be found: template data collection forms; data extracted from included studies; data used for all analyses; analytic code; any other materials used in the review. | **Availability of data and materials**.(Page 13 Line 349)  The datasets used and/or analyzed during the current study are available from the corresponding author on reasonable request. |
